# OPN promotes the aggressiveness of non-small-cell lung cancer cells through the activation of the RON tyrosine kinase

**DOI:** 10.1038/s41598-019-54843-2

**Published:** 2019-12-02

**Authors:** Chengcheng Hao, Yuxin Cui, Siyuan Chang, Jing Huang, Emily Birkin, Mu Hu, Xiuyi Zhi, Wenbin Li, Lijian Zhang, Shan Cheng, Wen G. Jiang

**Affiliations:** 1 Department of Biochemistry and Molecular Biology, School of Basic Medical Sciences, Capital Medical University, Beijing, 100069 China; 2 Beijing Key Laboratory of Cancer & Metastasis Research, Capital Medical University, Beijing, 100069 China; 3 Cardiff China Medical Research Collaborative, Cardiff University School of Medicine, Heath Park, Cardiff, CF14 4XN UK; 4 Department of Oncology, Beijing Shijitan Hospital, Capital Medical University, Beijing, China; 5 Department of Thoracic Surgery, Beijing Xuanwu Hospital, Capital Medical University, Beijing, China; 6 Department of Thoracic Surgery, Peking University School of Oncology and Beijing Cancer Hospital & Institute, Beijing, 100142 P.R. China

**Keywords:** Oncology, Molecular medicine

## Abstract

Osteopontin (OPN) is identified as a diagnostic and prognostic biomarker of tumor progression and metastasis. In non-small-cell lung cancer (NSCLC), the functions of OPN have not been well characterized. The current study sought to investigate the clinical implications of OPN expression in NSCLC and the role of OPN in the aggressiveness of the lung cancer cells. Using a proteomics approach, we identified that phospho-RON (p-RON) was one of the most remarkably up-regulated proteins in OPN-overexpressing cells. The levels of OPN and RON transcripts were unveiled as independent prognostic indicators of survival in NSCLC (p = 0.001). Higher levels of OPN, RON and p-RON proteins were observed in tumor tissues. Knock down of the OPN gene suppressed the migration and invasion abilities of the A549 lung cancer cells which endogenously expresses OPN. While ectopic expression of OPN in the SK-MES-1 lung cancer cells increased levels of cellular invasion and migration. In addition, these changes were accompanied by a phosphorylated activation of RON. Small-molecule inhibition of RON or siRNA silencing of RON significantly reduced OPN-induced migration and invasion of lung cancer cells and had an inhibitory effect on the OPN-mediated cell epithelial-mesenchymal transition. Our study suggests that in NSCLC, the aberrant expression of OPN can be considered as an independent survival indicator and is associated with disease progression. OPN plays a crucial role in promoting migration and invasion properties of lung cancer cells through its phosphorylation activation of the RON signaling pathway, implying its potential as a therapeutic target in the treatment of NSCLC.

## Introduction

Lung cancer is one of the most prevalent and lethal cancers worldwide. Non-small-cell lung cancer (NSCLC) accounts for approximately 80–90% of all lung cancer^1^. The prognosis of NSCLC is poor and it is estimated that the 5-year survival rate for NSCLC is less than 20%^2^. High morbidity and mortality of NSCLC are mainly related to the high-frequency of metastasis. Therefore, it is crucial to find the major contributors to tumor metastasis and elucidate their molecular mechanisms involved in NSCLC progression in order to identify new targets for the development of anti-tumor therapies and prevention strategies.

Osteopontin (OPN) is an extracellular matrix phosphoprotein secreted by a number of cell types and is implicated in a variety of biological functions including cell adhesion, migration, immune response, bone calcification and tumor progression^3–5^. It has been reported that the level of OPN correlates with tumor grade and prognosis in patients with the bladder, breast, prostate, and colon cancers^6–8^. Therefore OPN has been considered as a biomarker for tumor progression in many human tumors^9–11^. In NSCLC, evidence of OPN oncogenicity is sparse. It has recently been shown that elevated OPN levels in tumors and serum are associated with malignant neoplasia^12–14^. Also NSCLC cell lines that natively express OPN have greater metastatic potential and invasive behavior^5,15^, but the molecular pathways for OPN during tumorigenicity are otherwise not understood.

Recepteur d’Origine Nantais (RON), also known as the macrophage stimulating 1 receptor (MST1R), is mainly expressed in macrophages, osteoclasts, hematopoietic and epithelial cells^16–19^. As one member of the receptor tyrosine kinase (RTK) family, RON is involved in a series of biological functions such as immune response and wound healing^20,21^. Aberrant expression of RON is associated with tumor stage, poor prognosis and invasiveness in many cancer types^22^. It is known that RON functions as a mediator of tumor progression in some cancers^23,24^. Once activated by its ligand, macrophage-stimulating protein (MSP), RON promotes the invasive growth of cancer cells mainly through activation of several signaling pathways such as β-catenin, PLCγ, PI3K/AKT and MAPK *etc*.^25–27^. RON also plays a role in immunosuppression by regulating the properties of tumor-associated macrophages in the tumor microenvironment^28^. RON is highly expressed by osteoclasts and has been shown to participate in bone metastasis through mediating dysregulated bone absorption^29^. It is also known that the functions of RON in cells can be independent of MSP^30^. In lung cancer, recent studies indicate that RON may be a potent biomarker of tumor progression, metastasis and prognosis^31^. To date, however, the potential biological roles of RON in the tumorigenesis of NSCLC and downstream pathways have not been fully elucidated.

The current study sought to investigate the expression levels of OPN protein in tissues from surgical patients with NSCLC and to evaluate the association of this molecule with clinical features and outcome. We also examined the *in vitro* biological functions of OPN in human lung cancer cell lines (namely A549 and SK-MES-1) after gene knockdown and ectopic expression, respectively. Our protein microarray analysis data established the link between OPN expression and the activation of RON in lung cancer cells, which led us to further investigate the combined prognostic value of RON and the regulation of RON signaling pathways by OPN in the aggressiveness of NSCLC cells.

## Methods

### Human lung cancer specimens

For gene expression profile analysis, we obtained a cohort of lung cancer patients with long-term follow-up from Peking University Cancer Hospital from 2003 to 2011. The study was approved by local ethics committees (Peking University Cancer Hospital and Xuanwu Hospital of Capital Medical University Ethics Committees) and performed in accordance with guidelines established by the World Medical Association Declaration of Helsinki. Written consent was obtained from all patients.

We obtained seventy seven paired tumor and adjacent normal tissues from this cohort (n = 77). Clinical information of the patients for gene expression analysis is summarized in Table 1. The gene expression data from the cohort were analyzed after normalization using glyceraldehyde-3-phosphate desidrogenase (GAPDH) as an internal control.

**Table 1:** Expression of OPN and RON genes in tissue from lung cancer patients.

Characteristic		Number
**(*a*)**		
Type	Tumour	77
	Normal	77
Histology	Squamous	30
	Adenocarcinoma	41
	Small cell carcinoma	3
	others	3
Tumour staging	T-1	9
	T-2	43
	T-3	13
	T-4	12
Node status	N-0	36
	N-1	15
	N-2	26
Metastasis	M-0	77
	M-1	0
TNM	TNM1	24
	TNM2	18
	TNM3	35
	TNM4	0
Smoking history	No smoking	27
	Smoking	50
**Parameter**	**F value**	**p value**
**(*b*)**		
Differentiation	0.240	0.626
Embolism	0.000	0.993
Pleural invasion	0.170	0.682
T-stage	3.861	**0**.**054**
N-stage	9.277	**0**.**004**
TNM stage	8.098	**0**.**06**
Bone involvement	0.048	0.827
Smoking	0.532	0.469
OPN	11.620	**0**.**001**
RON	13.498	**0**.**001**

(*a*) The clinicopathological parameters of the lung cancer cohort for gene expression profiling. (*b*) Multivariate analysis of clinicopathological factors influencing overall survival.

For immunohistochemical (IHC) analysis, fresh frozen NSCLC tumor and paired adjacent normal tissues were obtained from twenty patients who received curative resection in Xuanwu Hospital of Capital Medical University from December 2011 to May 2014 (n = 20). These tissues were collected immediately after surgical resection and stored in the Tissue Bank of Cancer Institute of Capital Medical University. Ethical approval was provided by the Xuanwu Hospital of Capital Medical University Ethics Committees. Written consent was obtained from all patients.

### IHC staining

Frozen sections of NSCLC (n = 20) and paired adjacent normal lung tissues (n = 20) were cut at a thickness of 4 μm using a cryostat. The sections were first fixed with 4% paraformaldehyde. Endogenous peroxidase activity was blocked with 3% hydrogen peroxide for 30 min. Sections were incubated for 30 min in 5% goat serum albumin blocking solution and probed with monoclonal mouse anti-OPN primary antibody (1:200) (ab8448, Abcam, Cambridge, UK), rabbit anti-p-Ron (Tyr1238/Tyr1239) (1:200) (sc-22193, Santa Cruz, CA, USA), and rabbit anti-RON (EP1132Y)(1:200) (ab52927, Abcam, Cambridge, UK). A peroxidase conjugated goat anti-mouse immunoglobulin IgG was purchased from ZSGB Biotechnology (Beijing, China). The diaminobenzidine (DAB) chromogen (Cell Signal Technology, Danvers, MA, USA) was used for the coloration. Finally, the sections were observed under a microscope (BX43, Olympus, Tokyo, Japan).

For data analysis, slides from all 20 pairs of the specimens were analyzed independently by two observers using light microscopy. The percentage of carcinoma cells with cytoplasmic/membranous/nuclear staining was recorded and a total of 5 microscopic fields were randomly selected and captured. Staining for OPN was evaluated with Image-Pro Plus (IPP) v6.0 software (Media Cybernetics, USA). The strength of protein expression was calculated by the formulation of the IOD (Integrated option density)/Area.

### Plasmids and chemical reagents

The OPN cDNA was cloned into a GV147 vector by Genechem Co., Ltd. (Shanghai, China) to establish the OPN expression construct. The shRNA plasmid targeting OPN was constructed by Genechem using a GV248 vector and the targeting sequence is CCTGTGCCATACCAGTTAA. RON-targeting siRNA duplexes of 5′-GGAGUACUAUAGUGUUCAA-3′ and 5′-CCUGCUGGACACACUAAUUTT-3′ were purchased from Genepharma (Suzhou, China). A scrambled siRNA 5′-AAUUAGUGUGUCCAGCAGGTT-3′ was used as the siRNA control. MK8033, a novel and specific dual ATP competitive c–Met/Ron inhibitor, was obtained from MedChemExpress (MCE, New Jersey, USA).

### Reverse transcription-polymerase chain reaction (RT-PCR)

RNA extraction, reverse transcription (RT) and polymerase chain reaction were performed as described previously^32^. The pair of primers used for examining mRNA expression of OPN by PCR were 5′-TTGCAGTGATTTGCTTTTGC-3′ (sense) and 5′-GTCAATGGAGTCCTGGCTGT-3′ (antisense). PCR was performed under the following conditions: an initial denaturation at 94 °C for 15 seconds; 30 cycles of denaturation at 95 °C for 30 seconds, annealing at 58 °C for 30 seconds, and extension at 72 °C for 30 seconds; and a final extension at 72 °C for 5 minutes. A 305 bp PCR product was analyzed by Tris-acetate EDTA agarose (1% w/v) gel electrophoresis.

### Quantitative analysis of OPN and RON gene transcripts in lung tissues

Levels of gene transcripts for OPN and RON were determined using quantitative RT-PCR in a qPCR thermocycler (BioRad Icycler IQ5). The detection system was based on the Amplifluor Uniprimer^TM^ probe system, in which a FAM-tagged universal probe was used (Millipore, England). The primers for the gene transcripts were designed in such a way that each primer sits in a unique region of the gene target and in two separate exons. A Z-sequence (5′ actgaacctgaccgtaca 3′) was added to one of the primers and worked with the Universal probe to allow the formation of FAM-tagged amplicons. GAPDH gene transcripts were also quantified in the same cohort and used for the normalization. The primer pairs used for the respective gene transcripts were as follows: OPN (5′ agaagcagaatctcctagcc 3′ and 5′ actgaacctgaccgtacacatggtcatcatcatcttca 3′); RON (5′ catccacccagtgccaac 3′ and 5′ actgaacctgaccgtacaccacacagtcagccacag 3′); and GAPDH (5′ ctgagtacgtcgtggagtc 3′ and 5′ actgaacctgaccgtacacagagatgatgacccttttg 3′).

### Cell culture and transfection

Human lung adenocarcinoma A549 cells and squamous SK-MES-1 cells were obtained from the American Type Culture Collection (ATCC, Manassas, VA, USA). Cells were routinely cultured with Dulbecco’s modified Eagle medium (DMEM) supplemented with 10% fetal calf serum and 1× antibiotics. Cells were transfected with plasmids and siRNA by using Lipofectamine 3000 transfection reagent (L3000015, Invitrogen, USA). Stably transfected cells were selected by G418 for OPN overexpression or puromycin for OPN shRNA knockdown. After two weeks, we established the stable OPN-overexpressing/OPN-low expressing cell lines.

### Western blotting and antibodies

Western blotting was performed as described previously^33^. Briefly, cells were cultured and collected, and then lysed in a lysis buffer (NaCl 150 mM, Tris 50 mM, Sodium azide 0.02%, Sodium deoxycholate 0.5%, Triton X-100 1.5%, Aprotinin 1 μg/ml, Na_3_VO_4_ 5 mM, Leupeptin 1 μg/ml, PMSF 100 μg/ml, SDS 0.1%, DTT 100 μM). Equal amounts of protein were separated by SDS-PAGE and blotted onto PVDF membranes. The membrane was then probed with the respective primary antibodies and corresponding secondary antibodies. Anti-OPN, anti-RON (EP1132Y), anti-E-Cadherin, anti-N-Cadherin, anti-Twist and anti-β-Catenin antibodies were purchased from Abcam (Cambridge, UK). Anti-Slug, anti- Snail and anti-GAPDH antibodies were from Santa Cruz Biotechnology (California, USA). The human phospho-MSPR/Ron (Y1238/Y1239) antibody was from R&D Systems (Minnesota, USA). The blotted proteins were then detected with a Z-ECL chemiluminescence kit (Luminata Forte; Millipore, Hertfordshire, UK) and photographed using the G-Box gel documentation system (Syngene, Cambridge, UK).

### Scratch wound-healing migration assay

Cells were seeded to a 24-well plate to form a monolayer. Pipette tips were then used to scratch a line on the monolayer of each well. The gaps were captured under an inverted microscope at each time point as indicated during the incubation period. The distance of gap closure as a consequence of cellular migration was measured using ImageJ software (National Institutes of Health, http://rsbweb.nih.gov/ij/).

### Cell invasion assay

Transwell chambers, equipped with a 6.5 mm diameter polycarbonate filter insert (pore size 8 μm) (Becton Dickinson, Labware, Oxford, UK), were pre-coated with 50 μg/insert of Matrigel. Cells were seeded at a density of 2 × 10^4^ cells/insert. After incubation for 48 hours, the cells that invaded through the Matrigel were quantified using a crystal violet staining assay (Sigma-Aldrich).

### Electrical cell impedance sensing (ECIS) based cell migration assay

This method has been widely implemented by our laboratories and was outlined previously^34^. Briefly, 96-well W96E1 microarrays were used on the ECIS®Zθ (theta) instrument (Applied Biophysics Ltd, Troy, New Jersey, USA). Lung cancer cells were added to the wells of the array. Then confluent lung cancer monolayers in the arrays were electrically wounded (2000 mA for 20 seconds each), after which the migration of the cells was immediately tracked over a range of frequencies (1000–64000 Hz). All the experiments were conducted in triplicate. And the initial resistance reading of the SKMES-1 and A549 stable cell lines by the ECIS system was shown in Supporting Information Fig. S1.

### Kinex antibody microarray

At the end of the treatment as indicated, lung cancer cells in T75 flasks were washed twice in ice-cold PBS and then sonicated in standard Kinex lysis buffer (20 mM MOPS, 1% Triton X-100, 2 mM EGTA, 5 mM EDTA, 30 mM NaF, 60 mM β-glycerophosphate, 20 mM sodium pyrophosphate, 1 mM Na_3_VO_4_, 1 mM phenylmethylsulfonyl fluoride, 1× complete protease inhibitor mixture (Roche Applied Science), 1 mM DTT). The protein concentration was then quantified using fluorescamine protein-dye and the bovine serum albumin (BSA) was used as a standard. Approximately 100 μg of lysate protein samples were shipped in dry ice to Kinexus Bioinformatics Corporation (Vancouver, Canada) for Kinex™ KAM-880 Antibody Microarray service with 60% pan-specific antibodies and 40% phospho-specificantibodies. For the analysis of the Kinex protein microarray data, the normalized signal strength of protein isoforms and Z-ratio of sample comparison was used to identify the regulated candidate proteins. Z ratios were calculated by the division of the z-score difference from two groups by the standard deviation of the difference, and used as an indicator of differential expression of individual proteins in the group of OPN-overexpressing cells (threshold >1.5 or <−1.5).

### Statistical analysis

SPSS version 19.0 (SPSS, Inc., Chicago, IL, USA) was used for statistical analyses. The association of expression with clinical features was analyzed using the Mann-Whitney U test. The IHC results were assessed using the paired t-test. The Student’s t-test was used to analyze the cell functions with two groups, while the analysis of variance (ANOVA) was used to compare the data of multiple groups. A p-value < 0.05 was defined as statistically significant.

### Ethics approval and consent to participate

The study protocol was approved respectively by both Peking University Cancer Hospital and Xuanwu Hospital of Capital Medical University Ethics Committees. All of the participants signed an informed consent form.

## Results

### The expression level of RON has prognostic value as significant as that of OPN in patients with NSCLC

**Figure 1:**
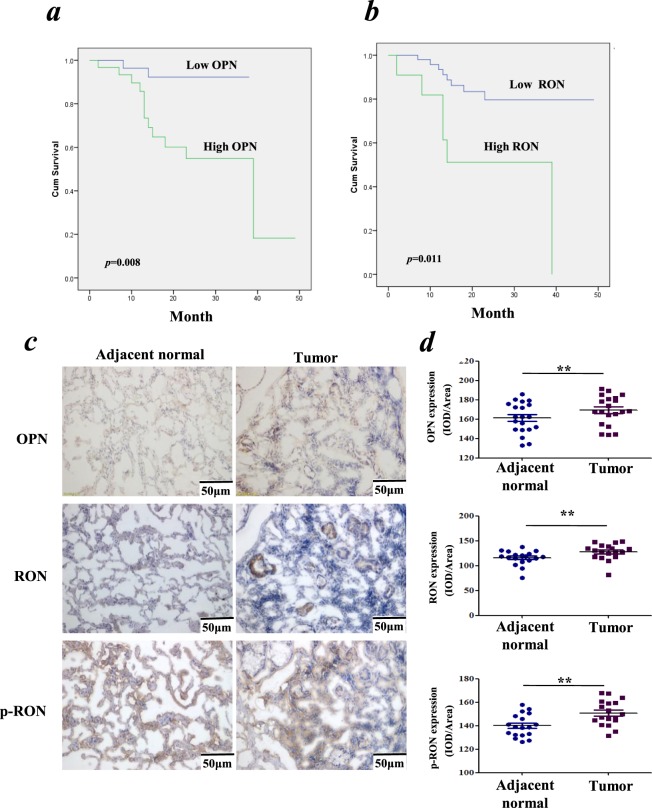
Association of OPN expression and RON activation in NSCLC. (**a**) The cumulative survival of lung cancer patients is positively associated with the expression of OPN. (**b**) The cumulative survival of lung cancer patients is positively associated with the expression of RON. (**c**) Representative images of IHC staining with OPN, RON and p-RON (Y1238 + Y1239), respectively, in NSCLC tissue sections, compared with matched adjacent normal tissue sections (SP × 200). (**d**) IHC staining respectively revealed the positive expression of OPN, RON and p-RON (Y1238 + Y1239) in NSCLC tissue sections, compared with matched adjacent normal tissue sections (n = 20).

To investigate the association of transcription level of the OPN with patient survival in lung cancer, we performed gene expression profile analysis in a lung cancer patient cohort which included 77 pairs of tissues, namely tumor tissues and adjacent normal lung tissues (Table 1a). As shown in Fig. 1a, Kaplan-Meier survival analysis indicated that poorer patient survival was associated with higher expression of the OPN gene (*p* = 0.008). In the same cohort, as shown in Fig. 1b, poor patient survival was positively associated with elevated expression of the RON gene (*p* = 0.011). Multivariate analysis of clinicopathological factors influencing overall survival suggested that RON, like OPN, was also an independent factor to predict overall survival of patients (*p* = 0.001), in line with the T-, N- or TNM stage (Table 1b). By IHC, our data indicated that higher levels of OPN, RON and p-RON (Y1238 + Y1239) were expressed in the NSCLC tissues compared with the paired adjacent normal tissues (*p* < 0.01, respectively) (Fig. 1c). In addition, RON and p-RON were mainly located on lung epithelial cell membranes and cytoplasm in adjacent normal tissues, while in cancer tissues, their expressions were mainly detected in the cytoplasm of lung cancer cells.

### OPN promoted the phosphorylated activation of RON in NSCLC

**Figure 2:**
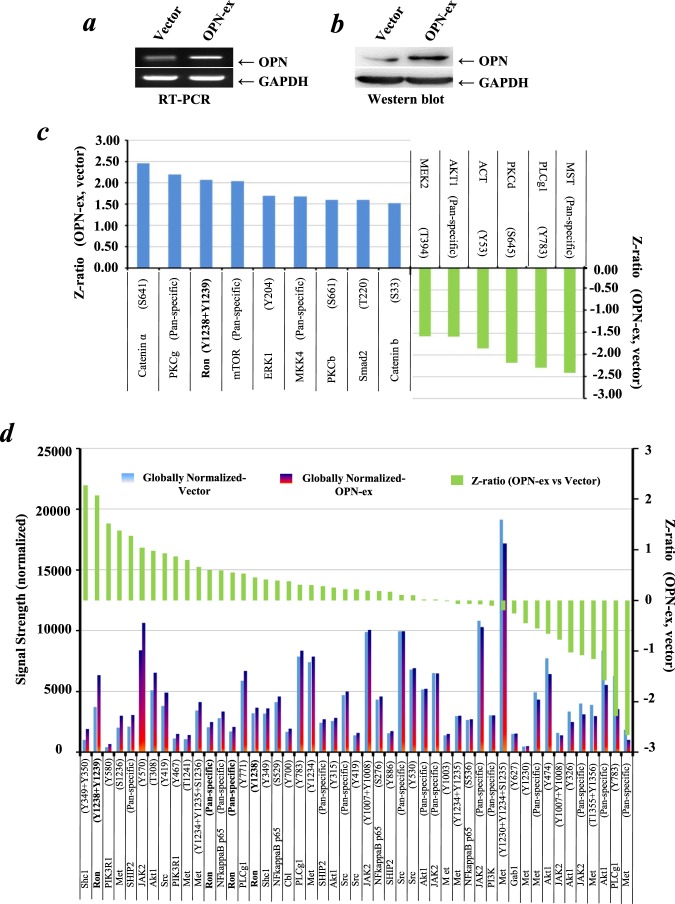
Profile analysis of signaling proteins in OPN-overexpression and control SK-MES-1 cells with the Kinex antibody microarray. (**a**) Verification of OPN overexpression in SK-MES-1 cells at a mRNA level by RT-PCR. (**b**) Verification of OPN overexpression in SK-MES-1 cells at a protein level by Western blotting. Western blotting developed using different antibodies or gels were sepreated by white space. (**c**) Lead proteins with significant change (at least Z ratio ± 1.5) in OPN-overexpressed SK-MES-1 cells were identified by the Kinex antibody microarray. (**d**) Profile of the RON-related signaling pathway proteins determined by the Kinex antibody microarray. Briefly, the signaling protein profiles of the cell lysate samples (labled as Vector and OPN-ex) were analyzed using the Kinex™ KAM-880 Antibody Microarray service Kinexus Bioinformatics Corporation (Vancouver, Canada) with 60% pan-specific antibodies and 40% phospho-specificantibodies. The expression level data are presented as globally-normalized signal strength of each individual protein isoforms in each sample. For fold change determination, Z Scores are calculated first by transforming the background-corrected spot intensity values for individual proteins after converting the globally-normalized mean to zero. As the transformation of z-scores is done before sample-to-sample comparison, it is therefore comparison-independent. Z ratios were then calculated by the division of the z-score differences of the two samples (i.e. OPN-ex substracted from Vector) by the standard deviation (SD) of all the differences for the comparision. The values of z ratios are presented as the level changes of individual proteins between the control and treated samples.

We then established the OPN overexpression stable cell lines using SK-MES-1 cells which have a relatively low level of endogenous OPN expression (Supporting Information Fig. S2). The stable cell lines were subjected to sequential validation using RT-PCR (Fig. 2a) and Western blot (Fig. 2b) following puromycin selection for two weeks. To explore the molecular mechanism of OPN effects in lung cancer cells, the Kinex protein microarray analysis was utilized to compare the signaling proteomics of OPN-overexpression and the control of SK-MES-1 cells. As shown in Fig. 2c, following ectopic overexpression of OPN in SK-MES-1 cells, fifteen proteins were identified to be altered significantly according to their Z-ratios. Based on this outcome, we found that phospho-RON (p-RON) was one of the most remarkably up-regulated proteins in OPN-overexpressing SK-MES-1 cells. In additional, the data from this protein microarray analysis enabled us to elucidate the levels of the RON-related signaling pathway checkpoint proteins as shown in Fig. 2d.

### Effects of OPN overexpression and knockdown on *in vitro* functions of NSCLC cell lines

**Figure 3:**
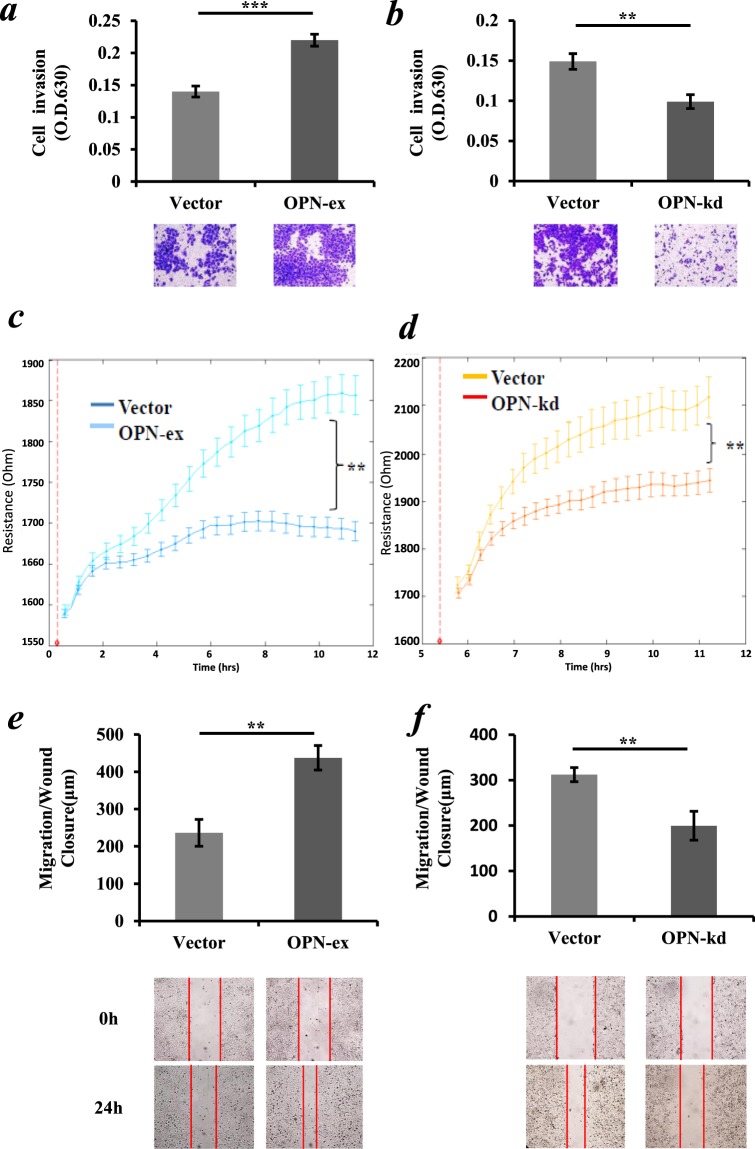
Effect of OPN expression level on the aggressive property of NSCLC cells. (**a**) A significant increase of *in vitro* Matrigel invasion in OPN-overexpressing SK-MES-1 cells. (**b**) Knockdown of OPN in A549 cells significantly reduced cellular Matrigel invasion. (**c**) OPN overexpression in SK-MES-1 cells increased cellular migration when assessed using ECIS after electric wounding (red dotted line), as indicated by resistance. (**d**) Knockdown of OPN markedly inhibited the post-wound migration capacity of A549 cells in the ECIS system which showed decreased resistance. (**e**) OPN overexpression in SK-MES-1 cells increased migration capacity after cultivation for 24 hours. (**f**) Knockdown of OPN significantly reduced cellular migration capacity of A549 cells. The results represent the mean values ± SD of three independent experiments. *p < 0.05, **p < 0.01.

Major malignant phenotypes of cancer cells including cell invasion and migration were evaluated first. As shown in Fig. 3a, ectopic overexpression of OPN promoted the transwell invasion of SK-MES-1 cells (*p* < 0.01). On the other hand, after transfection of the OPN-shRNA transgene into A549 cells with endogenous high OPN expression, the invasion property of A549 cells was significantly reduced (*p* < 0.01) (Fig. 3b). In addition, Ectopic overexpression of OPN in SK-MES-1 cells showed a remarkable increase in post-wound migration as indicated by the ECIS system and scratch wound-healing assay (*p* < 0.01, respectively) (Fig. 3c,e). In contrast, the knockdown of OPN expression resulted in a remarkable reduction in the migration ability of A549 cells (*p* < 0.01, respectively) (Fig. 3d,f).

### The altered EMT related gene expression profile by OPN was dependent on the phosphorylation and activation of RON

**Figure 4:**
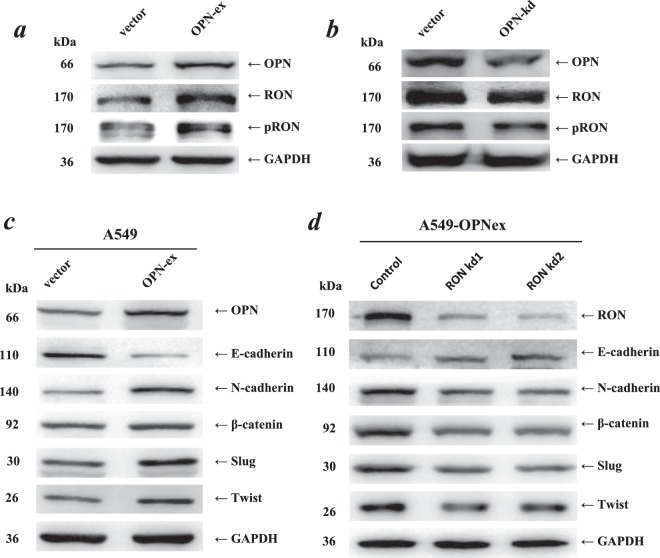
Alteration of the EMT-related protein levels by OPN was dependent on the phosphorylated activation of RON. (**a**,**b**) The levels of total RON and p-RON (Y1238 + Y1239) proteins were significantly increased in OPN-overexpressed A549 cells but decreased in A549 cells which were treated with OPN shRNA, as indicated by western blotting. (**c**,**d**) The expression of OPN, RON and EMT markers (E-cadherin, N-cadherin, β-catenin, Slug, Twist) in A549 cells. OPN downregulated E-cadherin expression and upregulated the expression of N-cadherin, β-catenin, Slug, Twist. The OPN-induced differential expression of the EMT markers was reversed following the knockdown of RON by siRNA1 and siRNA2. Western blotting developed using different antibodies or gels were sepreated by white space.

We then investigated whether OPN could promote the phosphorylated activation of RON in NSCLC using Western blotting. As shown in Fig. 4a,b, compared with those of the control groups, the levels of RON and p-RON were significantly increased in the OPN-expressing A549 cells but decreased after knockdown of OPN expression. E-cadherin gene, the typical epithelial-to-mesenchymal transition (EMT) molecular marker, was significantly downregulated in OPN-overexpressed A549 cells. Accordingly, another four typical EMT molecular markers (N-cadherin, β-catenin, Slug, and Twist) were upregulated in the OPN-overexpressed A549 cells (Fig. 4c). Knockdown of RON reversed the OPN-induced downregulation of E-cadherin and upregulation of N-cadherin, β-catenin, Slug and Twist (Fig. 4d). These results indicated that the promotion effects of OPN on cell migration and subsequent invasive abilities could be due to OPN-induced cell EMT which was dependent on the phosphorylated activation of RON signaling.

### The colocalization and interaction between OPN and RON in NSCLC cells

**Figure 5:**
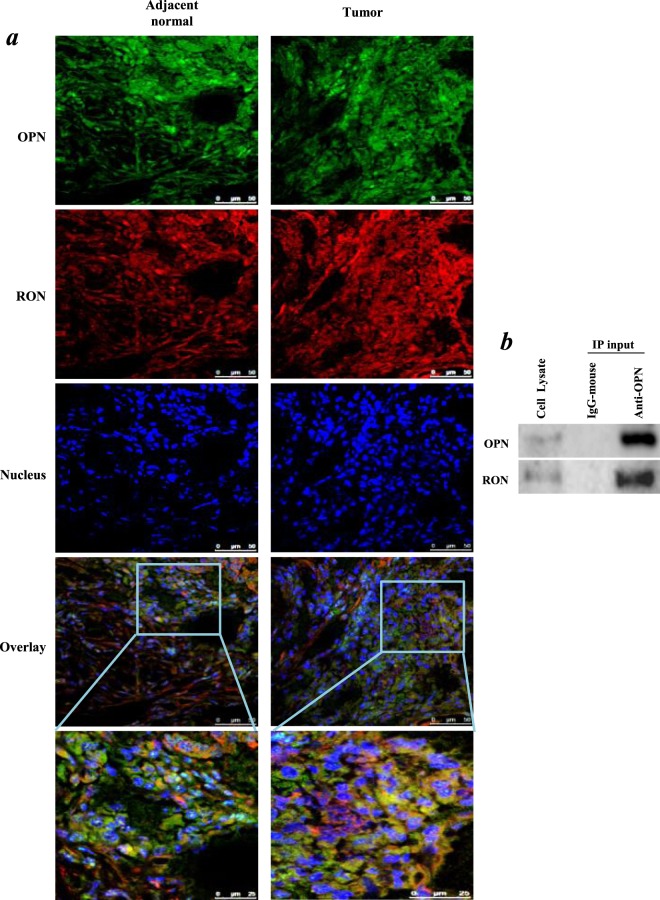
Co-localization and interaction of OPN and RON proteins. (**a**) OPN (shown in green) and RON (shown in red) were probed in lung carcinoma and matched adjacent normal tissue. Nuclei were stained with Hoechst dye 33258. Co-localization was investigated by confocal microscopy. (**b**) Immunoprecipitation (IP) of OPN from whole cell lysate of A549 cells. An irrelevant IgG was used as a control of the anti-OPN antibody during IP. Immunoprecipitated proteins and the whole cell lysate were then analyzed by western blotting. Western blotting developed using different antibodies or gels were sepreated by white space.

To visualize the distribution of OPN and RON in NSCLC, we performed the multi-colour immunofluorescence using NSCLC tissue sections. As shown in Fig. 5a, OPN was mainly located in the cytoplasm, while RON was stained both in the cytoplasm and membrane. Their co-localization was observed in the cytoplasm and strengthened with the increased distribution of RON in the cytoplasm of tumour cells. We further performed CO-IP assay in order to evaluate the interaction between OPN and RON proteins. As shown in Fig. 5b, RON was indeed pulled down together with OPN by the OPN antibody in A549 cells, whereas none of these two proteins was recovered when an irrelevant antibody (IgG) was used for IP, thus establishing the specificity of the assays. The Co-IP result indicated that there was physical interaction between OPN and RON in A549 cells.

### Secreted OPN promoted the malignant phenotypes of lung cancer cells through the mediation of the RON signaling pathway

**Figure 6:**
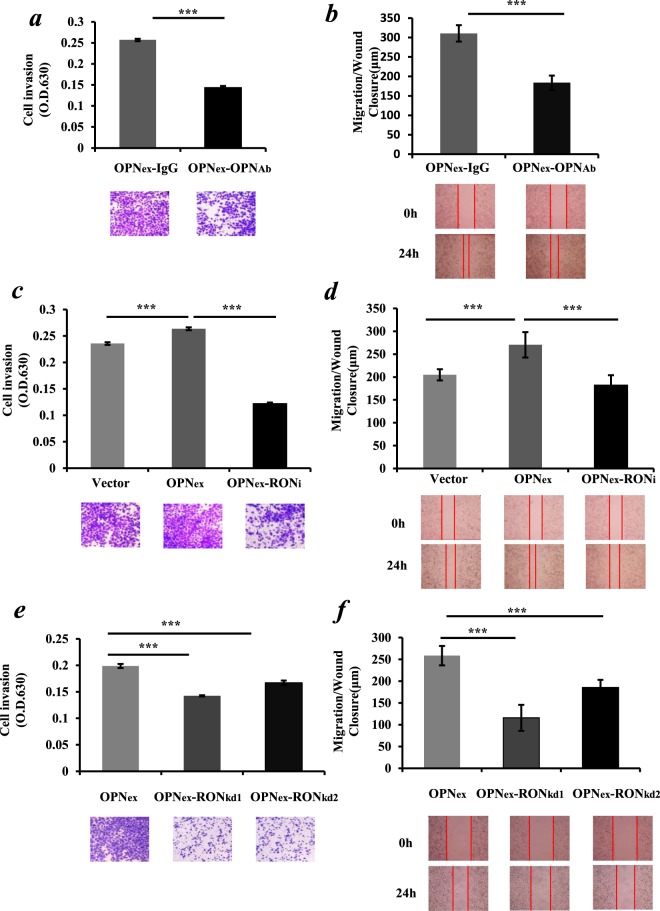
Secreted OPN promoted the malignant phenotypes of lung cancer cells mediated by the RON signaling pathway. (**a**,**b**) Functions of the OPN-overexpressing A549 cells including invasion and migration, respectively, were dramatically inhibited in the presence of OPN-neutralizing antibody (OPN-Ab) respectively compared to IgG-neutralizing antibody (IgG-Ab) control groups (*p* < 0.01). OPN-overexpressing A549 cells were treated with media containing 20 μg/ml of OPN-Ab and IgG-Ab, respectively, to block the secreted OPN protein. (**c**,**d**) The elevated levels of invasion and migration A549 cells by OPN overexpression were abolished after cells were exposed to 10 μM of the RON inhibitor MK8033 (RONi) for 24 hours compared to the control groups (p < 0.001). (**e**,**f**) knockdown of RON by siRNA (kd1 and kd2, respectively) in OPN overexpressing A549 cells significantly reduced the invasion and migration capacities of the cells, respectively. The results represent the mean values ± SD. **p < 0.01, ***p < 0.001.

An antibody neutralization assay was carried out in order to explore whether OPN-induced cell migration and invasion were mainly caused by secreted OPN. We supplemented OPN-neutralizing antibody and IgG-neutralizing antibody respectively to the media of the cultured OPN-overexpressing A549 cell line. As shown in Fig. 6a, The OPN-neutralizing antibody inhibited cell invasion by over 40% in OPN-overexpressing A549 cells, compared to the control groups with an IgG-neutralizing antibody in medium (*p* < 0.001). Likewise, the OPN-neutralizing antibody also inhibited cell migration by over 50% in OPN-overexpressing A549 cells compared to the IgG control (*p* < 0.01) (Fig. 6b). In order to further explore the influence of the RON signaling pathway on cell migration and invasion induced by OPN, we conducted the experiments with RON knockdown and exposure of the cells to MK803, a novel and specific Ron inhibitor. Compared to the control group, the invasion and migration of A549 cells in the OPN overexpression group were significantly enhanced, respectively (*p* < 0.001). However, after the addition of MK803, the invasion and migration abilities of the OPN-overexpressing A549 cells were significantly reduced, respectively (Fig. 6c,d. *p* < 0.001). Knockdown of RON by gene silencing also significantly reduced cell invasion (Fig. 6e) and the post-wound migration (Fig. 6f) capacity of OPN-overexpressing A549 cells (*p* < 0.001, respectively).

As the level of OPN expressin in the wild-type A549 cell line is intermediate, to verify the effect of secreted OPN in coniditoned medium on aggressiveness of lung cancer cells, we further conducted new assays in the presence of the tumor conditioned medium from the stable SK-MES-1 cell line with OPN overexpression. As shown in Supporting Information Fig. S3, after OPN overexpression, both the overexpression of OPN in cells (OPNex) and the conditioned medium (OPN CM) from the OPNex cells promoted the cellular invasion and migration (p < 0.001 vs con CM, respectively). However, when the RON inhibitor MK8033 (RONi) or the si-RON (RONkd1 and RONkd2) were applied, the induced cell migration and invasion by the secreted-OPN containing CM were eliminated in the SK-MES-1 cell line. The presence of OPN protein in the conditioned medium was confirmed by Western blot while several albumin/MMP-cleaved fragments of OPN protein in medium can also be observed (Supporting Information Fig. S4) as reported elsewhere^35,36^.

## Discussion

OPN is one of the known proteins considered to be associated with tumor genesis and progression in malignancies. It has been reported that OPN expression is upregulated in a variety of human carcinomas, including breast, prostate, lung and melanoma. And the expression levels of OPN are often closely related to clinical outcomes in cancer patients^37^. In the current study, we have shown that OPN is frequently over-expressed in both NSCLC tissue and plasma (Supporting information Table S1) which are mirrored well with each other. These findings support that OPN is a valuable indicator for diagnosis and prognosis in NSCLC patients. This is supported by previous studies which show that the elevated level of OPN plasma level in patients with NSCLC are reduced after resection or following platinum-based chemotherapy, but seem to upturn with recurrence^38,39^.

In order to further characterize this biomarker, we focus on the roles of OPN in the mediation of the malignant phenotype of NSCLC cells. Our results provide evidence that over-expression of OPN is linked to the elevation of invasion and migration of SK-MES-1 cells *in vitro*. In contrast, following genetic silencing of OPN, the properties of cellular invasion and migration in A549 cells are reduced. These results suggest a positive role of OPN in the aggressiveness of lung cancer cells.

NSCLC cell lines that natively express OPN have greater metastatic potential and invasive behaviour, but the molecular pathways for OPN during tumorigenicity are otherwise not understood. Our protein microarray study shows that RON and p-RON are both significantly increased in OPN-overexpressing SK-MES-1 cells. IHC results suggest that expression levels of OPN, RON and p-RON are dramatically elevated in NSCLC tissue when compared with paired adjacent normal tissue. Western blotting results also indicate that the levels of RON and p-RON are significantly increased in the OPN-expressing A549 cells but significantly decreased following OPN knockdown. Therefore, RON may play a vital role in participating in OPN-mediated tumorigenicity in lung cancer cells.

RON is a RTK belonging to the MET RTK family^40^. The specific ligand for RON is MSP, which is also known as HGF like protein (HGFL)^41^. Multiple mechanisms of aberrant RON signaling have been described previously. Classically, MSP binding to RON causes receptor tyrosine phosphorylation leading to up-regulation of RON catalytic activity and subsequent activation of downstream signaling molecules^42^. Alternatively, RON can be activated through other receptors by MSP-independent mechanisms^19^. Recent investigations have shown that the function of RTKs can be regulated by integrin-dependent cell adhesion to extracellular matrix (ECM) in the absence of a ligand^43^. ECM-induced integrin aggregation may lead to RON oligomerization and transphosphorylation, which depend on the kinase activity of RON itself. OPN, also known as an important member of the matricellular protein family, can bind integrins through the arginine-glycine-aspartate (RGD) domain^44,45^. Therefore, we speculate that the physical interaction between OPN and RON may be in a MSP-independent manner and is possibly mediated by integrins.

Kaplan-Meier survival analysis and multivariate analysis of clinicopathological factors suggest that the RON transcript expression, like OPN, is also an independent factor to predict the overall survival of patients. Increased RON expression is also found in OPN-overexpressing NSCLC tissues and cells. Therefore, it is possible that OPN induces the transcript expression of RON, and then elevates the level of p-RON. In seeking the relevance of the current findings, the putative transcription factor binding sites (TFBS) in RON promoter sequence have been identified in the Alggen–promo database (http://alggen.lsi.upc.es). In total 24 transcription factors are predicted to bind the RON promoter sequence and regulate transcription of the *RON* gene (Supporting Information Fig. S5). Several of them have been reported to be involved in the OPN regulated signal networks, such as NF-1^46^, p53^47^ and Sp1^48^.

**Figure 7:**
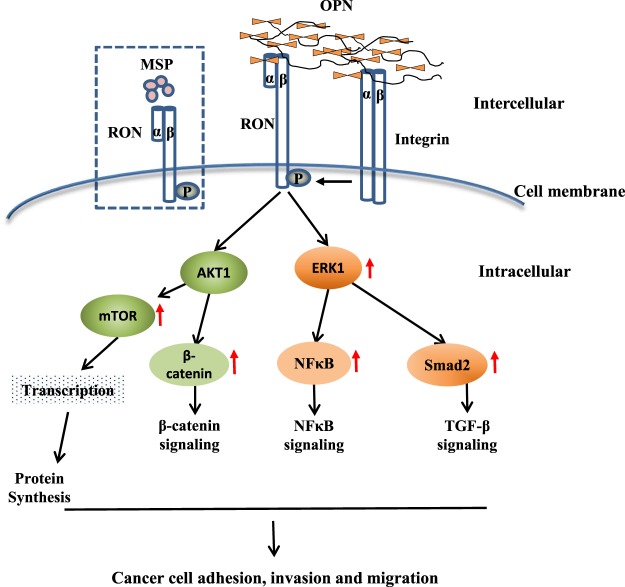
Schematic illustration of molecular mechanisms underlying the aggressiveness induced by the protein-protein interaction of OPN and RON in lung cancer cells. The changes of the relevant signaling check-point proteins were identified by the Kinex antibody array.

Our data confirm that the expression of OPN can induce RON receptor tyrosine phosphorylation, which could induce the subsequent activation of downstream signaling cascade molecules such as Catenin, ERK, Smad and NFκB and promote malignant phenotypes of lung cancer cells (schematically illustrated in Fig. 7). It has been reported that MSP-induced EMT relies on the phosphorylation and activation of RON and Erk1/2^43^. We show here that small molecule inhibition or gene silencing of RON significantly reduces OPN- overexpression-induced migration and invasion of lung cancer cells, and inhibits the OPN-induced cell EMT. This suggests that the RON signaling pathway participates in the OPN-induced malignant properties through mediating the EMT program in lung cancer cells. To the best of our knowledge, this is the first report describing the regulation of OPN on RON in lung cancer cells. Herein we provide evidence indicating the potential biological roles of OPN and RON in the progression of NSCLC, it might be interesting to further investigate the therapeutic potential of targeting the OPN/RON downstream signaling pathways in NSLC.

## Conclusion

In conclusion, our data indicate that higher levels of OPN are associated with poorer patient outcome in NSCLC. This study demonstrates for the first time that OPN exerts oncogenic functions in lung cancer cells through the modulation of the activated phosphorylation of RON. Inhibition of RON activity may provide therapeutic advantages for blocking OPN-promoted malignant phenotypes of cancer cells including migration, invasion and EMT. All these findings lay the foundation for the possible clinical application of OPN and RON, not only as biomarkers for diagnostic and prognostic purposes, but also as potential therapeutic targets in NSCLC.

### Novelty and impact

The clinical significance and molecular role of OPN in the progression of non-small-cell lung cancer (NSCLC) have not been well characterized. Here, the authors provide evidence that OPN and RON are both independent indicators of patient survival. They further find that OPN plays a crucial role in promoting aggresiveness of lung cancer cells through its phosphorylation activation of the RON signaling pathway, implying its potential as a therapeutic target in the treatment of NSCLC.

## Supplementary information

Supporting information Table S1, and Figure S1, S2, S3, S4 and S5.

## Data Availability

The datasets used and/or analysed during the current study are available from the corresponding author on reasonable request.
