# Supplementary material for: OPN promotes the aggressiveness of non-small-cell lung cancer cells through the activation of the RON tyrosine kinase
Source: Sci Rep. 2019 Dec 2;9:18101. doi: 10.1038/s41598-019-54843-2 (PMC6889187; doi:10.1038/s41598-019-54843-2)
Supplement: Supplementary file 1 — Supporting information Table S1, and Figure S1, S2, S3, S4 and S5. [file 41598_2019_54843_MOESM1_ESM.pdf]

OPN promotes the aggressiveness of non-small-cell lung cancer cells through the activation of the RON tyrosine kinase

Chengcheng Hao[a,b,c,d*], Yuxin Cui[c*], Siyuan Chang[a,b*], Jing Huang[a,b], Emily Birkin[c], Mu Hu[e], Xiuyi Zhi[e], Wenbin Li[d], Lijian Zhang[f], Shan Cheng[a,b,§], Wen G Jiang[a,b,c, §]

a Department of Biochemistry and Molecular Biology, School of Basic Medical Sciences, Capital Medical University, Beijing 100069, China.

b Beijing Key Laboratory of Cancer & Metastasis Research, Capital Medical University, Beijing100069, China.

c Cardiff China Medical Research Collaborative, Cardiff University School of Medicine, Heath Park, Cardiff CF14 4XN UK.

d Department of oncology, Beijing Shijitan Hospital, Capital Medical University, Beijing, China

e Department of Thoracic Surgery, Beijing Xuanwu Hospital, Capital Medical University, Beijing, China

f Department of Thoracic Surgery, Peking University School of Oncology and Beijing Cancer Hospital & Institute, Beijing 100142, P.R. China

* These authors contributed equally to this work.

[§]**Correspondence Author:** Wen G Jiang, Cardiff China Medical Research Collaborative, Cardiff University School of Medicine, Heath Park, Cardiff CF14 4XN UK. Email: jiangw@cf.ac.uk; Shan Cheng, Department of Biochemistry and Molecular Biology, School of Basic Medical Sciences, Capital Medical University, Beijing 100069, China. Email: chengs@ccmu.edu.cn

Materials and Methods for the Supplemental data

Human Lung Cancer Blood Specimens

In order to evaluate the level of secreted OPN in blood, 75 plasma samples of NSCLC patients were enrolled from 2012 to 2013. The relevant information is provided in Suppl. Table 1. The study was approved by a local ethics committee (Xuanwu Hospital of Capital Medical University Ethics Committees) and performed in accordance with guidelines established by the World Medical Association Declaration of Helsinki.

ELISA

Blood samples for OPN measurement were collected in tubes with EDTA anticoagulant and plasma separations were performed by centrifugation of the blood samples (room temperature, 3000 rpm for 15 min). Finally, supernatants were divided into aliquots and stored at -80°C until

the day of the assay. OPN in plasma was quantified using a Human Osteopontin ELISA Kit (CSB-E08392h, CUSABIO, USA) following the manufacturer's instructions. The accuracy of these assays was tested against plasma samples with known concentrations of the tested analytes.

Transcription Factor Binding Sites analysis

The sequence of the RON gene promoter, including the 2000 bp upstream sequence and 500 bp downstream sequence from the transcriptional starting site (TSS), was downloaded from the UCSC Genome Browser (http://genome.ucsc.edu/). The putative transcription factor binding sites (TFBS) in the RON promoter sequence was identified in the Alggen–promo database (http://alggen.lsi.upc.es). Maximum matrix dissimilarity rate was set at 5%.

Supporting information Table S1. Levels of plasma OPN protein from the lung cancer patients. Correlation of the plasma OPN protein level and the clinicopathological parameters was analyzed using the Kruskal–Wallis test. A value of $p < 0.05$ was considered statistically significant.

Parameter		Patients		
		Number	Median plasma OPN concentration（ng/mL）	P-value
**Sample**				**0.015**
	Normal	10	7.58	
	Tumour	75	23.13	
**Gender**				0.482
	Male	45	24.69	
	Female	30	22.51	
**Histology**				0.093
	Adenocarcinoma	34	20.94	
	Squamous cell carcinoma	17	11.56	
**Smoking history**				0.573
	Yes	40	19.69	
	No	35	20.63	
**TNM**				0.057
	Ⅰ	9	10.00	
	Ⅱ	2	27.26	
	Ⅲ	15	25.16	
	Ⅳ	28	22.82	
**T**				0.157
	T1	9	14.38	
	T2	18	19.07	
	T3	10	28.13	
	T4	17	15.63	
**N**				0.617
	N0	11	14.38	
	N1	2	19.85	
	N2	25	18.13	
	N3	10	24.53	
**Metastasis**				0.775
	Yes	28	19.83	
	No	47	17.13	

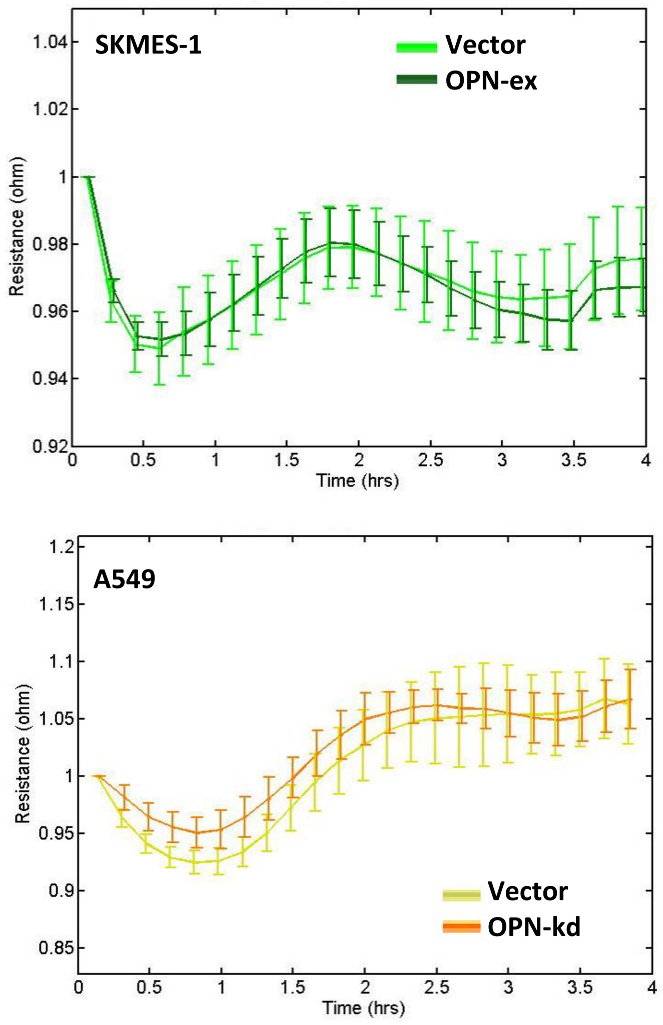

Supporting Information Fig. S1. Initial resistance reading of the SKMES-1 and A549 stable cell lines by the ECIS system. Data are shown as means±sd, n=3.

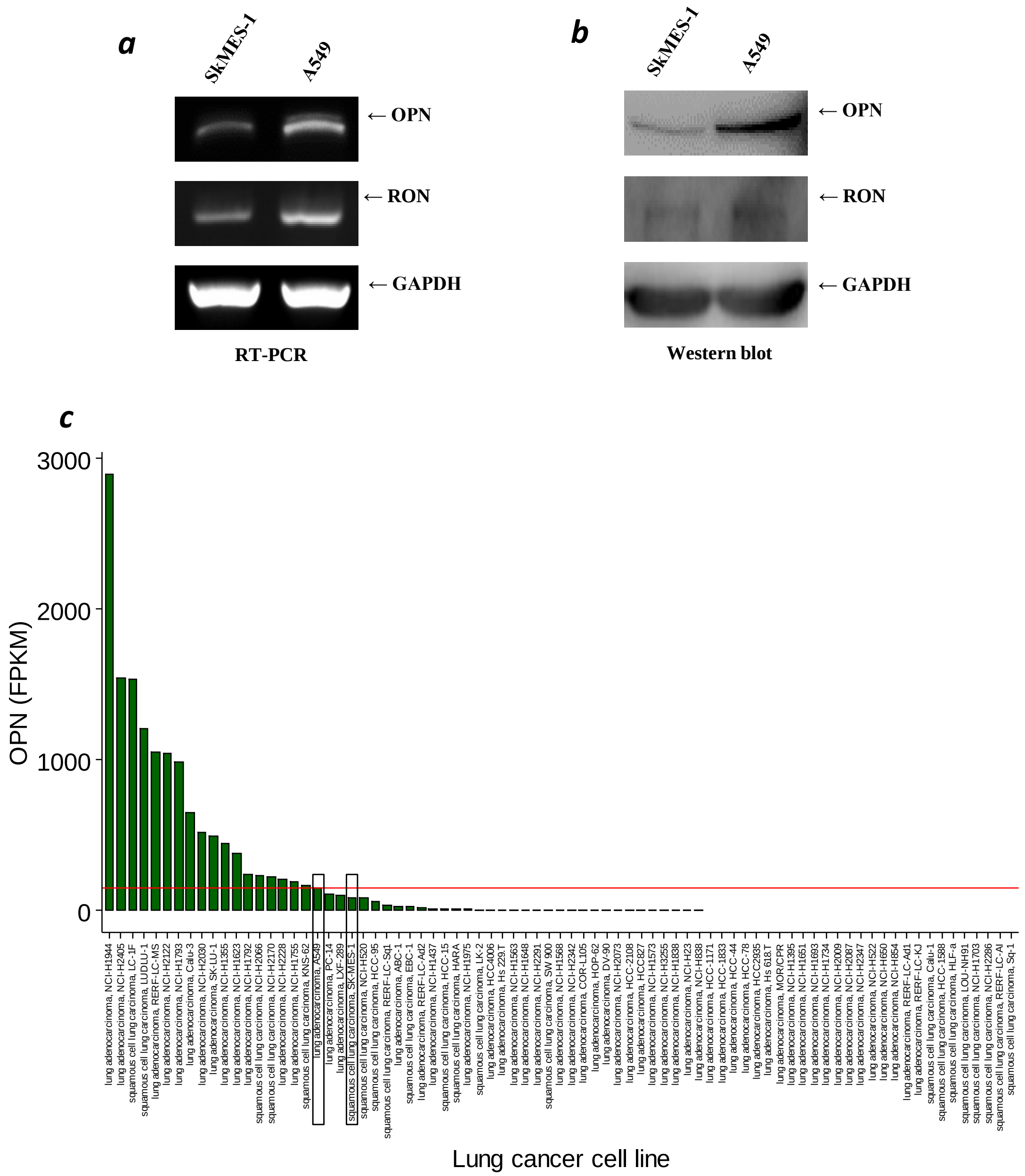

Supporting Information Fig. S2. The basal level of OPN and RON expression in A549 and SKMES-1 cell lines. (A) mRNA expression indicated by RT-PCR. (B) Protein expression indicated by Western blot. (C) mRNA expression profile of OPN in LUAD and LUSC cell lines in the database of RNA-seq of human cancer cell lines from the Broad-Novartis Cancer Cell Line Encyclopedia (http://www.broadinstitute.org/ccle).

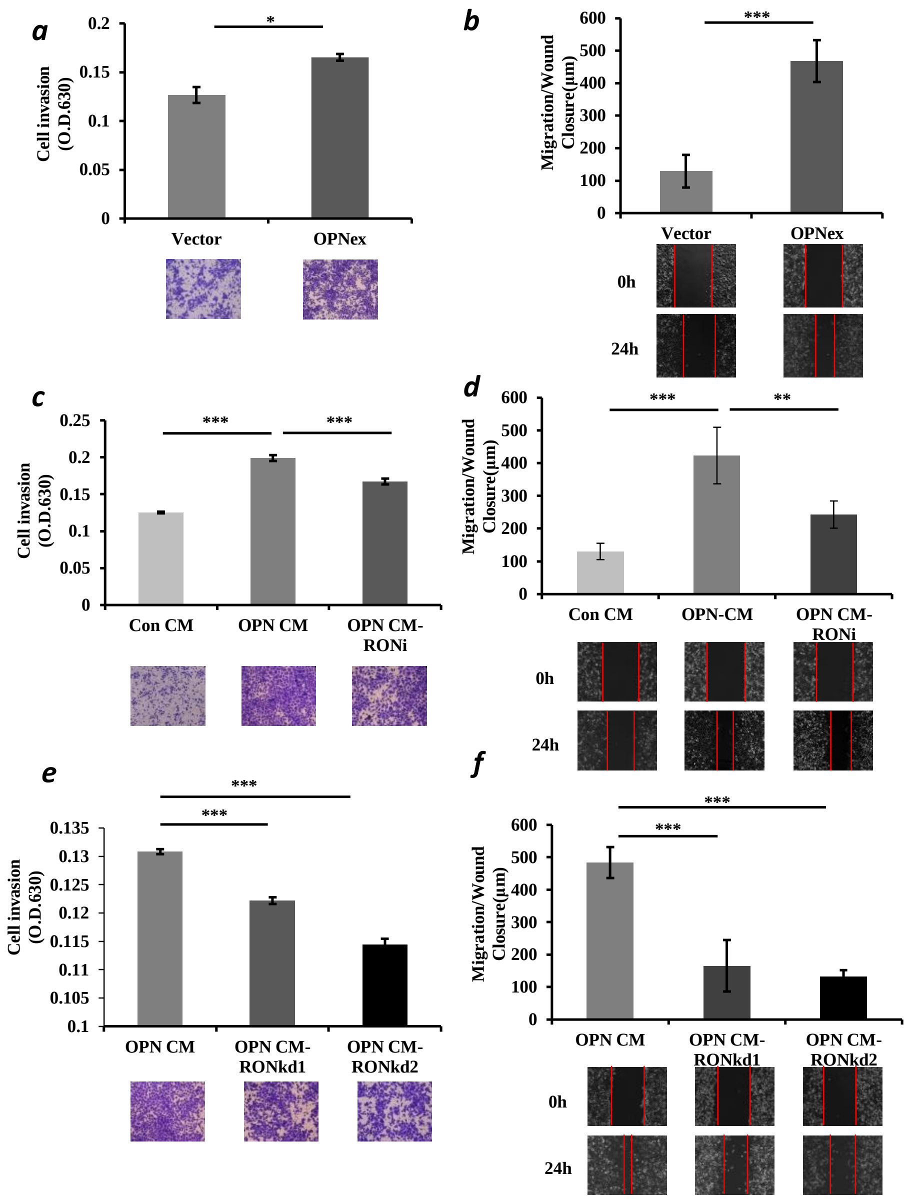

Supporting Information Fig. S3. Secreted OPN promoted the malignant phenotypes of SK-MES-1 cells mediated by the RON signaling pathway. (a) and (b) Functions of the OPN-overexpressing SK-MES-1 cells including invasion and migration, respectively. (c) and (d) The elevated levels of invasion and migration SK-MES-1 cells induced by OPN conditioned medium (OPN CM) were abolished after cells were exposed to 10 µM of the RON inhibitor MK8033 (RONi) for 24 hours compared to the control groups. (e) and (f) Knockdown of RON by siRNA (kd1 and kd2, respectively) in SK-MES-1 cells significantly reduced the invasion and migration induced by OPN CM, respectively. The results represent the mean values ± SD. *P<0.05, ** p<0.01, *** p<0.001.

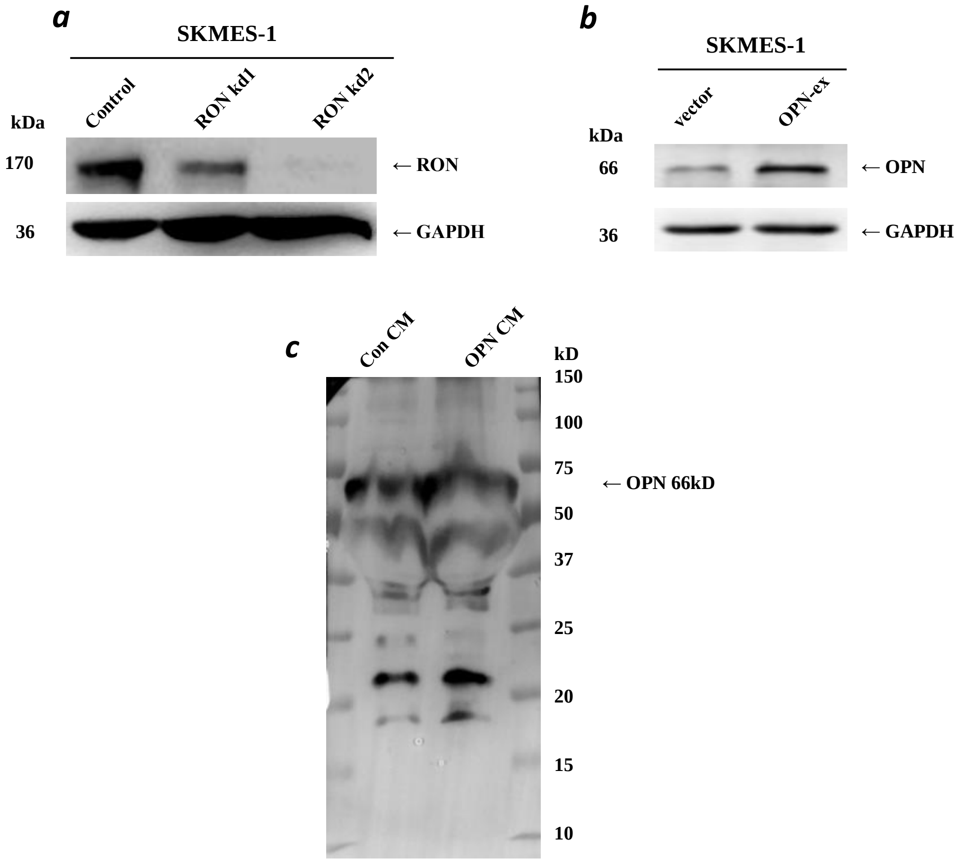

Supporting Information Fig. S4. Validation of RON knockdown, OPN overexpression and OPN conditioned medium in SK-MES-1. (a) Validation of RON knockdown by siRNA (kd1 and kd2, respectively) in SK-MES-1 cells at a protein level by Western blotting (b) Validation of OPN overexpression in SK-MES-1 cells at a protein level by Western blotting. (c) Validation of OPN content in the conditioned medium collected from culture medium of OPN overexpressing SK-MES-1 cells (OPN CM) and vector control cells (Con CM) respectively by Western blotting. Total OPN is approximately 66kD. In culture medium there are also several cleaved fragments due to the presence of albumin or MMP, which was as expected.

a

>RON promoter_1.

AGGGCCTGGGCTAGGCCAAGCCTTCCTCGCGGCCCCGCCCCCACGGCCCGACTCCGCCCGCC
CAGCCCGGCGCCCTCGGGTCGGCTGAGCGCTAAGCGCCAGTGTACAGCGGCGGCTGGGGC
GGCAGGTGAGGCGGCTGGGGCGTTGCTGTCGTGCGTCCGCAGGCGTCAGGTGCTCAGACCC
GAGGGCCGGGAAGGGATT

>RON promoter_2.

GCCAATTCTGCTTTGGAAAAGGAGTGTCTTTTATGTCACCTCAGGGCACAGTATCAACCCTGC
ACTGGGTGTGGCCGCCCCCACTTCCATTTCCTCTTTCAGTCTTCAGGTGCTCATGGAGCTCCA
GGCTGGAAGGAGGGGGAGTCCTGCACTGGGGATGGAGGAAGTGAAGTATCCAGAGTAAAC
TGTGCTGGGGTGAGT

b

0 GR-alpha [T00337]	1 AP-2alphaA [T00035]	2 NFI/CTF [T00094]	3 TFII-I [T00824]	4 STAT4 [T01577]	5 c-Ets-1 [T00112]	6 Elk-1 [T00250]	7 Pax-5 [T00070]
8 p53 [T00671]	9 WT1 [T00899]	10 E2F-1 [T01542]	11 Sp1 [T00759]	12 ETF [T00270]	13 C/EBPbeta [T00581]	14 c-Jun [T00133]	15 RXR-alpha [T01345]
16 GR-beta [T01920]	17 USF2 [T00878]	18 ATF3 [T01313]	19 FOXP3 [T04280]	20 T3R-beta1 [T00851]	21 ENKTF-1 [T00255]	22 c-Ets-2 [T00113]	23 NF-1 [T00539]

c

1	10	20	30	40	50	60	70	80	90	100	110	120	130	140	150	160	170	180	190	200	210
RON promoter_1.	0 1 7 8	0 1 2 13 21 23	0 3 4 5 6 7 8 9 10 22	7 8 9 10 11 12	7 8 10	7 8 10 11 12	7 8 12	0 12 15		21	10	7 8 9 10 11 12	0 1 10 17 20	7 8 9 10 12	13 19	10	0 1 14 17 18	12 15	0 5 6 7 8	0 3 4 16	
RON promoter_2.	2 13 16	0 3 4 5 13 23	3	0 14 18 20	0 7 8	0 13 15 19	15	7 8 9 10 11 12 21	3 4 5 6 16	0 22	0 17		0 1 5 6	0 3 4	0 3	0 3 6 22	3 4 5	3	19	15 20	

Supporting Information Fig. S5. Putative transcription factor binding sites (TFBS) in the RON promoter sequence. The prediction of the transcription factors which bind the RON promoter sequence was performed using the Alggen–promo database (http://alggen.lsi.upc.es). (a) The sequence of the two promoters of RON in the range of -100 to 100. (b) The predicted transcription factors which bind the RON promoter sequence. (c) The location of the binding sites of the predicted transcription factor in the RON promoters.
